# Psychological Distress and Coping Mechanisms Among Women Diagnosed With Breast Cancer: A Cross-Sectional Study of Anxiety, Depression, and Social Support

**DOI:** 10.7759/cureus.81831

**Published:** 2025-04-07

**Authors:** Sher Bano, Uchenna Chidinma Ibeji, Usman Jamil, Muhammad Saqlain, Maleeha Zulfiqar, Mahnoor Saeed, Obando Cabezas Maria Leonor, Syeda Maryam Zehra Zaidi, Javed Iqbal, Syed Muhammad Ali, Steve Paulson John, Abdulrahman AlQaderi

**Affiliations:** 1 Clinical Psychology, Brain Wave Research Center, Islamabad, PAK; 2 Internal Medicine, Uyun Al Jawa General Hospital, Uyun Al Jawa, SAU; 3 Internal Medicine, CMH Kharian Medical College, Islamabad, PAK; 4 General Surgery, Nishtar Hospital, Multan, PAK; 5 Medicine and Surgery, Barking, Havering and Redbridge University Hospitals NHS Trust (BHRUT), London, GBR; 6 Internal Medicine, Kabir Medical College, Peshawar, PAK; 7 General Surgery, The Robótic Global Surgical Society, Sherbrooke, CAN; 8 Medicine, Jinnah Medical and Dental College, Karachi, PAK; 9 Nursing, Hamad General Hospital, Doha, QAT; 10 Surgery, Weill-Cornell Medical School, Doha, QAT; 11 Acute Care Surgery, Hamad General Hospital, Doha, QAT; 12 Surgery, Qatar University College of Medicine, Doha, QAT; 13 Internal Medicine, Thumbay University Hospital, Dubai, ARE; 14 Medicine, RAK Medical and Health Sciences University, Ras Al-Khaimah, ARE

**Keywords:** anxiety, breast cancer, coping mechanisms, depression, psychological distress, social support

## Abstract

Introduction

Breast cancer is one of the most prevalent malignancies and a leading cause of cancer-related mortality among women. Beyond its physical impact, it significantly affects mental health, leading to anxiety, depression, and psychological distress. Coping mechanisms and social support play a crucial role in emotional resilience; however, limited research has examined these factors in Pakistani women. This study investigates the relationship between psychological distress, coping strategies, and social support among women diagnosed with breast cancer.

Method

This cross-sectional study included 100 women (N = 100, 100% female) diagnosed with breast cancer, recruited from private hospitals and clinics. Standardized instruments assessed generalized anxiety (Generalized Anxiety Disorder 7-item scale (GAD-7)), depression (Patient Health Questionnaire-9 (PHQ-9) 9-item scale), coping strategies (Brief Coping Orientation to Problems Experienced (COPE) Inventory 28-item scale), and social support (Multidimensional Scale of Perceived Social Support (MSPSS) 12-item scale). Data analysis was conducted using IBM SPSS Statistics for Windows, Version 26 (Released 2019; IBM Corp., Armonk, New York, United States), employing correlation analysis and group comparisons based on education, employment, and chemotherapy history.

Results

Higher generalized anxiety levels correlated with increased use of coping mechanisms (r = 0.133, p < 0.01), while perceived social support was positively associated with coping responses (r = 0.130, p < 0.01). Participants receiving chemotherapy reported higher social support (p = 0.016, Cohen’s d = 0.71), whereas non-recipients exhibited more significant depression (p = 0.001, Cohen’s d = 0.75).

Conclusion

Findings highlight the psychological burden of breast cancer and the critical role of coping strategies and social support. Tailored interventions are crucial for enhancing mental well-being, coping efficacy, and treatment outcomes, particularly among vulnerable groups.

## Introduction

Breast cancer remains the most commonly diagnosed cancer and the leading cause of cancer-related death among women globally. According to the World Health Organization, over 2.3 million women were diagnosed with breast cancer in 2020, with approximately 685,000 deaths reported worldwide [1]. In Pakistan, breast cancer accounts for nearly 14% of all cancer cases, with one in every nine women at risk of developing the disease during their lifetime, making it the highest incidence among all cancers in Pakistani women [2]. Breast cancer diagnosis creates both physical complications and psychological burdens that result in extreme distress, depression, and severe anxiety in patients [3]. The emotional state of distress produces symptoms that combine anxiety with depression and emotional turmoil, which disrupts usual functioning and life quality. Women with breast cancer face different levels of psychological distress, which intensifies because of anxiety regarding their prognosis and treatment side effects and body-related concerns and changes to their personal, family, and social roles [4,5].

Breast cancer patients commonly suffer from depression and anxiety, which together represent the primary psychological disorders affecting this population. Research shows that anxiety disorders develop in 20-50% of female breast cancer patients, but clinical depression affects 15-30% of this population [6,7]. Psychological disorders have adverse impacts on medication adherence, treatment outcomes, and life expectancy, underscoring the importance of early detection and intervention practices [8].

Research shows that social support emerges as an essential tool in coping, which affects cancer patients’ psychological adjustment and emotional strength. Research studies demonstrate that women with breast cancer who receive high levels of social support achieve better psychological results and show reduced depression symptoms, besides enjoying superior life quality [9]. The lack of sufficient social support leads to adverse outcomes by increasing psychological distress and weakening coping abilities, which in turn result in poor treatment adherence and negatively affect both disease prognosis and patient survival rates [10].

The development of psychological interventions and supportive care strategies requires a complete comprehension of how breast cancer patients handle their situations psychologically. The coping mechanisms people adopt can be categorized into two general groups: problem-focused approaches and emotion-focused approaches. People who use problem-focused coping choose to tackle their distressing situations actively by seeking information and practicing actions to control and reduce the original threats. The methods of emotion-focused coping include emotional response management, such as obtaining support and practicing relaxation and positive reframing, as well as avoidance strategies [11]. The use of effective coping methods leads women with breast cancer to maintain better emotional stability and overcome the challenges of their condition while experiencing lower psychological distress [12].

Scientific investigations focusing on cancer patients' psychological distress and coping approaches exist abundantly across the world, but specific research on Pakistani female patients is scarce. Patients' psychological outcomes and coping methods are heavily influenced by unique cultural features, socioeconomic status, and differences in the healthcare system, which require thorough examination within their local environments. The research fills a knowledge gap through assessments of breast cancer patients’ psychological distress, particularly understanding anxiety and depression levels, and their social networks and coping behaviors in Pakistan. The findings from this research help medical practitioners while directing policymakers toward developing cultural adaptations in their interventions to enhance both the mental health and total well-being of breast cancer patients.

Rationale and objectives

Breast cancer functions as a significant medical challenge worldwide because it delivers powerful consequences to women’s physical condition and mental states. A breast cancer diagnosis leads to substantial emotional turmoil that produces anxiety and depression and worsens patients’ quality of life as well as decreases their ability to follow treatment plans and affects how their cancer progresses. The provision of comprehensive cancer care requires complete knowledge about how affected women experience their mental health symptoms and their methods for handling emotional strain.

Multiple studies show that anxiety and depression frequently occur as psychological comorbidities in breast cancer patients, thus demanding systematic research investigating their coping behaviors along with existing social support structures. People diagnosed with cancer need coping mechanisms, which range from obtaining social and emotional help to processing issues and avoiding certain activities because these behaviors affect both mental resilience and treatment adjustment. The research consensus confirms that social support is a protective factor that reduces intense emotional distress, helping patients achieve better recovery outcomes.

Global research shows widespread progress, yet regional differences persist, highlighting the importance of research on cultural specificities. The artistic and social dynamics alongside economic forces in Pakistan shape how women experience and manage their difficulties during breast cancer. Research focused on psychological distress, coping strategies, and social support within these patients will help understand how cultural influences shape mental health management in breast cancer patients.

The purpose of this research investigation includes an analysis of generalized anxiety along with depression, alongside coping strategies and social support among female breast cancer patients. Research findings will support healthcare providers to create psychosocial interventions fitting the cultural needs of breast cancer patients, thus improving mental health alongside their entire recovery process.

## Materials and methods

This cross-sectional study explored the relationship between depression, coping mechanisms, and social support among 100 women diagnosed with breast cancer (N = 100, 100% female). Participants were purposively selected from various private hospitals and clinics. Purposive sampling was used to ensure that participants met specific inclusion criteria relevant to the study objectives. The inclusion criteria required participants to be adult females aged 18 or older, diagnosed with breast cancer, capable of understanding the study questionnaires, and willing to provide informed consent. The exclusion criteria ruled out individuals with a history of psychiatric disorders unrelated to breast cancer, those with cognitive impairments affecting their ability to complete the questionnaires, or individuals who declined to provide informed consent.

The sample size (N = 100) was initially calculated using the WHO sample size calculator, considering a 95% confidence level and a prevalence of 0.8. However, due to time and resource constraints, the study was conducted with 100 participants, ensuring representation of diverse demographic and clinical characteristics. Data collection was conducted through structured questionnaires between October 2024 and February 2025. Standardized instruments were utilized for assessment. The study utilized standardized psychological scales to measure the variables. Generalized anxiety was assessed using the Generalized Anxiety Disorder 7-item scale (GAD-7), developed by Spitzer et al. in 2006 (Appendix A). This scale comprises seven items scored on a 4-point Likert scale (0 = "not at all" to 3 = "nearly every day"). Total scores range from 0 to 21, with severity cutoffs at 5 (mild), 10 (moderate), and 15 (severe) [13]. Depression severity was measured using the Patient Health Questionnaire-9 (PHQ-9) (Appendix B) developed by Kroenke et al. in 2001. It consists of nine items rated on a similar 4-point scale, with score interpretation as follows: 5-9 (mild), 10-14 (moderate), 15-19 (moderately severe), and 20-27 (severe depression) [14]. Coping strategies were assessed using the Brief Coping Orientation to Problems Experienced (COPE) Inventory (Appendix C) developed by Carver in 1997. It comprises 28 items, divided into 14 subscales (two items each), rated on a 4-point Likert scale ranging from 1 (“I haven’t been doing this at all”) to 4 (“I’ve been doing this a lot”) [15]. Perceived social support was measured using the Multidimensional Scale of Perceived Social Support (MSPSS) (Appendix D) developed by Zimet et al. in 1988. It comprises 12 items distributed across three subscales - family, friends, and significant others - each rated on a 7-point Likert scale. Higher scores indicate more substantial perceived social support [16].

All the instruments used in this study have been previously validated and demonstrated acceptable to excellent reliability. The GAD-7 [13] has shown good internal consistency with a Cronbach’s alpha of 0.89, indicating strong reliability in measuring generalized anxiety. The PHQ-9 [14], used to assess depressive symptoms, also demonstrates high internal consistency with a Cronbach’s alpha of 0.89. The Brief COPE Inventory [15], which evaluates various coping strategies, has reported Cronbach’s alpha values ranging from 0.50 to 0.90 across its 14 subscales, reflecting variable but acceptable reliability for different coping domains. The MSPSS [16] has demonstrated excellent internal consistency, with a total scale Cronbach’s alpha ranging from 0.88 to 0.94 and subscale values ranging from 0.85 to 0.91. These psychometric properties support the use of these instruments for assessing psychological constructs in the present study population.

The collected data were analyzed using IBM SPSS Statistics for Windows, Version 26 (Released 2019; IBM Corp., Armonk, New York, United States). Descriptive statistics were used to summarize demographic variables, including means and standard deviations for continuous data and frequencies and percentages for categorical data. Inferential statistics included Pearson’s correlation analysis to examine the relationships between generalized anxiety, depression, social support, and coping strategies. Independent sample t-tests were conducted to compare scores between patients who received chemotherapy and those who did not. One-way ANOVA was used to analyze differences across educational and employment groups. The threshold for statistical significance was set at p < 0.05. Cronbach’s alpha values were computed to assess the internal consistency of all standardized scales used in the study.

Ethical approval for the study was granted by the Institutional Review Board (IRB-2024-0036) of the Brain Wave and Research Center, Islamabad, Pakistan. Participants were fully informed about the study’s objectives and assured that their confidentiality, privacy, and anonymity would be strictly protected. The collected data were used exclusively for academic purposes, ensuring participants’ autonomy and dignity were upheld throughout the research process.

## Results

Table 1 showcases the demographic data for study (N = 100) participants, including their age brackets, educational achievement, employment status, marital status, household income level, cancer stage, time since diagnosis, the distance to healthcare facilities, and visit frequency. Fifty-eight percent of participants were 18-30, while the average participant age was 30 (SD = 7.5). Regarding education, 16% of participants had primary education, 32% completed secondary school, 24% had undergraduate degrees, 16% were graduates, and 12% had no formal education. Thirty-two percent of respondents were work-at-home individuals, while participants living 5-10 kilometers from healthcare facilities comprised 65% of the sample group, thereby affecting their access to medical care. Thirty percent of participants received a stage 3 cancer diagnosis while participating in the study. Participants living 5-10 kilometers from healthcare facilities comprised 65% of the sample group, thereby affecting their access to medical care.

**Table 1:** Demographic characteristics of participants km: kilometers; mean age = 30 years (SD: 7.5) Cancer staging: Stage 0 = carcinoma in situ; Stage I-IV = increasing severity; Duration of diagnosis refers to the time since cancer diagnosis - household income in Pakistani Rupee (PKR). Distance to healthcare facilities is self-reported. Visit frequency indicates medical consultation regularity.

Variable	Frequency (f)	Percentage
Age (years)		
18-30	58	58
31-40	39	39
41-50	3	3
Education level		
Primary School	24	24
Secondary School	24	24
Undergraduate	24	24
Graduate/postgraduate	16	16
No formal education	12	12
Employment status		
Employed	12	12
Unemployed	12	12
Self-employed	27	27
Homeworker	32	32
Retired	17	17
Marital status		
Single	27	27
Married	49	49
Widowed	19	19
Divorced/separated	5	5
Household income		
20,000-50,000	20	20
51,000-100,000	21	21
101,000-200,000	40	40
Above 200,000	19	19
Cancer stage		
Stage 0	7	7
Stage 1	22	22
Stage 2	18	18
Stage 3	30	30
Stage 4	23	23
Duration of diagnosis		
Less than 6 months	7	7
6-12 months	18	18
1-2 years	34	34
2-5 years	30	30
More than 5 years	11	11
Distance to healthcare facility		
Less than 5 km	23	23
5-10 km	65	65
More than 10 km	12	12
Visit frequency		
Weekly	12	12
Monthly	39	39
Every few months	26	26
Rarely	8	8

Table 2 presents the intercorrelations between key study variables: generalized anxiety, depression, coping strategies, and perceived social support. The data show a significant positive correlation between generalized anxiety and coping strategies (r = 0.133, p < 0.01), suggesting that women experiencing higher anxiety symptoms tend to engage more frequently in various coping mechanisms.

**Table 2:** Intercorrelations between study variable * p < 0.01 considered significant COPE: Coping Orientation to Problems Experienced

Variable	1	2	3	4
Generalized anxiety (Generalized Anxiety Disorder 7 (GAD-7))	-	-	-	-
Coping strategies (Brief COPE Inventory)	0.133^*^	-	-	-
Social support (Multidimensional Scale of Perceived Social Support (MSPSS))	0.090^*^	0.130^*^	-	-
Depression (Patient Health Questionnaire-9 (PHQ-9))	0.042^*^	0.335^*^	-0.079^*^	-

Similarly, generalized anxiety also has a weak but significant positive correlation with perceived social support (r = 0.090, p < 0.01), indicating that individuals with higher anxiety levels perceive slightly greater support from their social environment. Moreover, a moderate positive correlation between coping strategies and perceived social support (r = 0.130, p < 0.01) reveals that individuals who actively use coping strategies also tend to report stronger social support.

The correlation between depression and coping strategies is also significant (r = 0.335, p < 0.01), implying that individuals experiencing more depressive symptoms tend to engage more in coping behaviors, possibly as a response to emotional strain. Interestingly, depression shows a weak negative correlation with perceived social support (r = -0.079, p < 0.01), suggesting that individuals with higher depressive symptoms may perceive less support from their surroundings.

These findings collectively highlight the complex and dynamic relationship between depression and adaptive behaviors in breast cancer patients. Increased depression, alongside anxiety, appears to influence the way patients seek support and employ coping strategies. These associations underscore the importance of integrating psychological care into cancer treatment plans.

Table 3 demonstrates the assessment of study variables through independent sample t-tests comparing participants who received chemotherapy versus those who did not. Participants showed comparable anxiety levels regarding generalized anxiety disorder (GAD) (t = 0.516, p = 0.607) and similar Brief COPE coping strategy scores (t = -0.236, p = 0.814) irrespective of their chemotherapy status. Recipient acceptance of chemotherapy led to higher perceived social support scores according to the MSPSS (t = 2.447, p = 0.016, Cohen's d = 0.71). The Patient Health Questionnaire data demonstrated critical variations (t = -3.359, p = 0.001, Cohen's d = 0.75) because non-chemotherapy patients showed higher depression scores. The 95% CI helps clarify an estimated area that contains potential authentic differences between tested groups. The data demonstrate that patients receiving chemotherapy perceived additional social support, whereas patients not on chemotherapy showed rising depression levels.

**Table 3:** Comparison among variables (chemotherapy) M: mean; SD: standard deviation; t: t-test value; p-value: significance level; CI 95%: 95% confidence interval; LL: lower limit; UL: upper limit; Cohen's d: effect size; COPE: Coping Orientation to Problems Experienced Independent sample t-tests compared study variables between participants receiving chemotherapy (Yes) and those not receiving chemotherapy (No). Significant differences were observed for the Multidimensional Scale of Perceived Social Support (p = 0.016) and the Patient Health Questionnaire (p = 0.001).

	Yes		No				CI 95%		
Variable					t	p-value			Cohen’s D
	M	SD	M	SD			LL	UL	
Generalized Anxiety (Generalized Anxiety Disorder 7 (GAD-7))	18.0	2.6	17.7	2.2	0.516	0.607	-0.87	1.48	-
Coping strategies (Brief COPE Inventory)	77.8	6.3	78.0	5.1	-0.236	0.814	-3.13	2.46	-
Social support (Multidimensional Scale of Perceived Social Support (MSPSS))	42.7	6.9	38.9	4.9	2.447	0.016	0.71	6.77	0.63
Depression (Patient Health Questionnaire-9 (PHQ-9))	24.1	2.4	26.0	2.7	-3.359	0.001	-3.13	-0.80	0.75

Table 4 displays results from a one-way ANOVA analysis showing variable differences across educational settings. Students with different education levels exhibited different coping strategies according to the Brief COPE results (F(4,95) = 4.36, p < 0.01, η² = 0.16). Participants who did not receive regular education demonstrated the highest Brief COPE score outcomes (M = 82.3, SD = 4.8), but undergraduate and graduate individuals scored the lowest (M = 75.7, SD = 6.1, M = 75.4, SD = 5.0, respectively). The different levels of education did not create significant differences in GAD scores (F = 1.71, p ≥ 0.05) or MSPSS results (F = 2.07, p ≥ 0.05) or Patient Health Questionnaire total scores (F = 1.77, p ≥ 0.05). This indicates that anxiety levels and social support perceptions, as well as psychological distress, remained comparable between educational groups. The research shows that educational factors affect students' coping, but psychological variables show similar patterns between different educational groups.

**Table 4:** Comparison of variables (educational level) M: mean; SD: standard deviation; F: ANOVA F-statistic; η²: effect size; COPE: Coping Orientation to Problems Experienced A one-way ANOVA was conducted to compare study variables across different educational levels. A significant difference was found for Brief COPE (F(4,95) = 4.36, p < 0.01, η² = 0.16), indicating variations in coping strategies based on education level. The symbol (*) indicates statistical significance at p < 0.01. No significant differences were observed for generalized anxiety disorder, Multidimensional Scale of Perceived Social Support, or Patient Health Questionnaire scores.

Variable	Primary	Secondary	Undergraduate	Graduate	No education	F (4,95)	η^2^
	M ± SD	M ± SD	M ± SD	M ± SD	M ± SD		
Generalized Anxiety (Generalized Anxiety Disorder 7 (GAD-7))	18.5 ± 2.6	17.1 ± 2.9	18.4 ± 1.9	18.3 ± 2.6	17.1 ± 1.9	1.71	-
Coping strategies (Brief COPE Inventory)	77.3 ± 5.8	79.8 ± 5.7	75.7 ± 6.1	75.4 ± 5.0	82.3 ± 4.8	4.36^*^	0.16
Social support (Multidimensional Scale of Perceived Social Support (MSPSS))	41.7 ± 6.2	43.3 ± 6.7	39.1 ± 6.4	44.4 ± 7.2	40.7 ± 6.4	2.07	-
Depression (Patient Health Questionnaire-9 (PHQ-9))	24.3 ± 2.9	24.8 ± 2.5	24.5 ± 2.3	23.8 ± 2.4	26.3 ± 2.9	1.77	-

The one-way ANOVA analysis in Table 5 shows how study variables differ across employment categories. The data showed that employment status led to statistically significant differences in Brief COPE measurement results with a small effect size (F(4,95) = 2.41, p < 0.01, η² = 0.09). The participants who worked as employees received the highest Brief COPE score rating (M = 80.0, SD = 6.4), yet self-employed and unemployed workers scored significantly lower (M = 76.3, SD = 4.7; M = 76.0, SD = 5.2). Studies revealed no substantial differences in GAD (F = 0.57, p ≥ 0.05) scores as well as MSPSS scores (F = 0.96, p ≥ 0.05) and Patient Health Questionnaire results (F = 1.08, p ≥ 0.05) across different employment types. Thus, generalized anxiety levels, perceived social support, and depression remained similar across employment groups. The study results demonstrate that employment status affects stress management, but psychological variables remain equal between groups.

**Table 5:** Comparison of variables (employment) M: mean; SD: standard deviation; F: ANOVA F-statistic; η²: effect size; COPE: Coping Orientation to Problems Experienced A one-way ANOVA was conducted to compare study variables across different employment statuses. A significant difference was observed for Brief COPE (F(4,95) = 2.41, p < 0.01, η² = 0.09), suggesting variations in coping strategies among employment groups. The symbol (*) indicates statistical significance at p < 0.01. No significant differences were found for generalized anxiety disorder, Multidimensional Scale of Perceived Social Support, or Patient Health Questionnaire scores.

Variable	Employed	Unemployed	Self-employed	Homeworker	Retired	F (4,95)	η^2^
	M ± SD	M ± SD	M ± SD	M ± SD	M ± SD		
Generalized Anxiety (Generalized Anxiety Disorder 7 (GAD-7))	17.1 ± 1.7	17.6 ± 2.9	17.9 ± 2.2	18.2 ± 3.0	18.1 ± 2.1	0.57	-
Coping strategies (Brief COPE Inventory)	80.0 ± 6.4	76.3 ± 4.7	76.0 ± 5.2	79.8 ± 6.5	76.5 ± 5.7	2.41*	0.09
Social support (Multidimensional Scale of Perceived Social Support (MSPSS))	40.9 ± 6.9	39.2 ± 4.9	43.2 ± 5.7	42.4 ± 7.9	40.8 ± 6.3	0.96	-
Depression (Patient Health Questionnaire-9 (PHQ-9))	24.8 ± 2.9	25.3 ± 2.2	23.7 ± 2.8	24.9 ± 2.5	24.7 ± 2.6	1.08	-

Table 6 displays descriptive statistics and chi-square assessments of demographic variables and employment status as they relate to visit frequency. The research discovered that education level correlated significantly with employment status through the χ² analysis, which produced a p-value of 0.004 (χ² = 34.7). Education level failed to create a noticeable relationship with medical consultation frequency since the chi-square value reached 7.09 while maintaining a p-value of 0.851. The analysis indicated that participants' employment status did not demonstrate a direct link to how far they resided from healthcare facilities because of the low p-value of 0.270, along with a χ² value of 9.93. Education levels seem to affect employment status, yet demographic aspects related to healthcare accessibility and visit frequency do not change according to educational attainment.

**Table 6:** Descriptive statistics of demographic variables f: frequency; p: significance level; χ²: chi-square statistic Chi-square tests were conducted to examine associations between demographic variables, employment status, and visit frequency. A significant association was found between education level and employment status (p = 0.004, χ² = 34.7), indicating differences in employment distribution based on education. No significant associations were observed for education level and visit frequency (p = 0.851, χ² = 7.09) or distance to healthcare facility and employment status (p = 0.270, χ² = 9.93).

Variables	f	Employed	Un-employed	Self-employed	Homeworker	Retired	p	x^2^	Weekly	Monthly	Every few months	Rarely	p	x^2^
Education level	-	-	-	-	-	-	-	-	-	-	-	-	-	-
Primary	24	4	5	9	5	1	0.004	34.7	8	8	6	2	0.851	7.09
Secondary	24	1	2	10	8	3	-	-	8	10	3	3	-	-
Under-graduate	24	2	4	3	10	5	-	-	5	8	9	2	-	-
Postgraduate	16	0	0	3	6	7	-	-	3	7	5	1	-	-
No education	12	5	1	2	3	1	-	-	3	6	3	0	-	-
Distance to a healthcare facility	-	-	-	-	-	-	-	-	-	-	-	-	-	8.99
Less than 5 km	23	6	3	7	5	2	0.270	9.93	10	9	4	0	0.174	-
5-10 km	65	5	6	18	23	13	-	-	16	26	17	6	-	-
More than 10 km	12	1	3	2	4	2	-	-	1	4	5	2	-	-

## Discussion

Breast cancer is not only a physical health concern but also a significant psychological burden that impacts mental well-being and alters both coping mechanisms and perceived social support networks. This study explored how women psychologically responded to their diagnosis and how they managed coping and support processes. The findings highlight the influence of demographic characteristics, treatment stages, and socioeconomic conditions on psychological adjustment. The results indicate a strong correlation between depression and both coping strategies and perceived social support, mainly due to its association with generalized anxiety levels. These findings emphasize the need for specific psychological intervention strategies. The subsequent discussion is based on a thorough analysis of the study’s statistical outcomes.

The research data demonstrated a statistically significant yet minimally strong relationship between GAD and coping strategies, which produced an r-value of 0.133 with p < 0.01 significance. The findings of [17] showed that anxiety as a trigger causes patients with chronic illnesses to adopt either beneficial or detrimental coping approaches. The study found that subjects with good social support networks tended to employ effective coping mechanisms, as indicated by a positive correlation between these variables (r = 0.130, p < 0.01). Social support perception shows a negative connection to depression (r = -0.079, p < 0.01) while demonstrating a positive relationship with coping mechanisms (r = 0.130, p < 0.01). The research illustrates how depression combines with coping methods and social structure in cancer treatment patients to show why social networks reduce distress levels.

Patients participating in chemotherapy exhibited higher levels of social support perception than those not undergoing chemotherapy (p = 0.016, Cohen’s d = 0.71). Healthcare professionals, family members, and cancer support groups establish organized support networks that contribute to this difference [18]. The absence of medical treatment, such as chemotherapy, created higher levels of psychological distress among patients (p = 0.001, Cohen’s d = 0.75), presumably because they perceive themselves as more uncertain about their future since they lack active medical intervention. Earlier research has already shown that monitored and supported chemotherapy patients maintain better emotional stability, yet non-chemotherapy patients struggle to maintain emotional balance [19]. Psychological interventions need to aim precisely at non-chemotherapy patients because they experience more significant emotional distress as they receive minimal structured care.

Those who didn’t receive any educational training demonstrated the highest Brief COPE scores (M = 82.3, SD = 4.8) compared to other educational levels (p < 0.01, η² = 0.16). Evidence from this research indicates that people with less formal education tend to build resilient coping strategies due to stress. The evaluation of education levels did not yield any noteworthy differences in anxiety levels (p > 0.05), social support perceptions (p > 0.05), or depression measurements (p > 0.05). Observations diverge from existing findings, showing that educated populations demonstrate improved mental well-being [20]. The study results indicate that a cancer diagnosis for the breast creates similar psychological effects for both highly educated and less educated patients.

Employment status significantly impacted coping mechanisms (p < 0.01, η² = 0.09), as people in employment roles scored higher on the Brief COPE (M = 80.0, SD = 6.4) compared to self-employed and unemployed respondents, whose scores were M = 76.3 (SD = 4.7) and M = 76.0 (SD = 5.2), respectively. Workers receive better access to resources and structured settings, which help them manage several issues. The research indicated no meaningful distinctions in the levels of anxiety (p ≥ 0.05), perceived social support (p ≥ 0.05), and depression (p ≥ 0.05) between those employed, self-employed, and unemployed individuals. Scientific studies confirm that employment strengthens coping approaches but fails to minimize mental health problems in patients who have cancer [18]. According to the study findings, employment provides organized methods to control stress, yet it does not protect people from cancer-related emotional distress.

The analysis revealed that education level has a direct impact on employment status, as employment rates increased among patients with higher education levels (p = 0.004, χ² = 34.7). However, the results indicated that education level did not significantly influence the frequency of visits to healthcare facilities (p = 0.851, χ² = 7.09) or the distance from healthcare services (p = 0.270, χ² = 9.93). These findings suggest that while education improves job opportunities for breast cancer patients, it does not necessarily translate into better access to healthcare facilities. Moreover, the data challenge previous assumptions linking employment stability with improved healthcare access [21].

The study presents important findings regarding adaptive psychological responses and the influence of social support among women diagnosed with breast cancer. A significant strength of this study lies in its comprehensive assessment of psychosocial variables, which enhanced the accuracy and interpretative value of the findings. Validated tools, such as the GAD-7 and PHQ-9, ensured reliable measurements. However, the study has certain limitations. Firstly, the subdomains of the Brief COPE and MSPSS scales were not analyzed separately, which may have limited the depth of understanding regarding specific coping strategies and sources of social support. Additionally, since GAD-7 is a screening tool for generalized anxiety symptoms, it cannot be interpreted as a diagnostic tool for GAD. The study’s cross-sectional design restricts causal inferences, making it difficult to determine whether changes in anxiety, depression, or coping strategies precede or follow one another. Self-reported data may have introduced response bias. To strengthen future research, longitudinal study designs should be considered to monitor changes over time, and intervention-based testing should be utilized to evaluate the long-term effectiveness of psychological support programs. Furthermore, exploring the correlation between the subdomains of coping and social support could offer deeper insights into the psychological adaptation process in this population.

## Conclusions

This study reveals an extensive interaction network between depression and coping mechanisms, together with social support, which affects women dealing with breast cancer. Social support levels were higher among chemotherapy patients, yet their depression remained lower than non-chemotherapy patients, who showed higher emotional distress. The participants’ educational attainment, together with employment status, influenced their coping strategies, yet these factors did not affect either their perceived social support systems or their experience of distress. The study results demonstrate why targeted psychological support programs must target non-chemotherapy patients because they appear to face more significant distress risks. Developed research programs must prioritize support methods that help patients develop stronger coping skills and social relationships to improve their mental health during breast cancer treatments.

## Appendices

Appendix A

**Table 7:** GAD-7 anxiety GAD-7: Generalized Anxiety Disorder 7 Reference: [13]

Item	Response
Feeling nervous, anxious, or on edge	-
Not being able to stop or control worrying	-
Worrying too much about different things	-
Trouble relaxing	-
Being so restless that it is hard to sit still	-
Becoming easily annoyed or irritable	-
Feeling afraid, as if something awful might happen	-

Appendix B 

**Table 8:** PHQ-9 patient depression questionnaire PHQ-9: Patient Health Questionnaire-9 Reference: [14]

Items	Response
Little interest or pleasure in doing things.	
Feeling down, depressed, or hopeless	
Trouble falling or staying asleep, or sleeping too much	
Feeling tired or having little energy	
Poor appetite or overeating	
Feeling bad about yourself or that you are a failure or have let yourself or your family down	
Trouble concentrating on things, such as reading the newspaper or watching television	
Moving or speaking so slowly that other people could have noticed. Or the opposite being so fidgety or restless that you have been moving around a lot more than usual	
Thoughts that you would be better off dead, or of hurting yourself	

Appendix C 

**Table 9:** Brief - Coping Orientation to Problems Experienced Inventory (Brief-COPE) Reference: [15]

Items	Response
I’ve been turning to work or other activities to take my mind off things.	
I've been concentrating my efforts on doing something about the situation I'm in.	
I’ve been saying to myself “this isn’t real.	
I’ve been using alcohol or other drugs to make myself feel better	
I’ve been getting emotional support from others	
I’ve been giving up trying to deal with it.	
I’ve been taking action to try to make the situation better.	
I’ve been refusing to believe that it has happened.	
I’ve been saying things to let my unpleasant feelings escape.	
I’ve been getting help and advice from other people	
I’ve been using alcohol or other drugs to help me get through it	
I’ve been trying to see it in a different light, to make it seem more positive.	
I’ve been criticizing myself.	
I’ve been trying to come up with a strategy about what to do	
I’ve been getting comfort and understanding from someone.	
I’ve been giving up the attempt to cope	
I’ve been looking for something good in what is happening.	
I’ve been making jokes about it.	
I’ve been doing something to think about it less, such as 19 going to movies, watching TV, reading, daydreaming, sleeping, or shopping	
I’ve been accepting the reality of the fact that it has happened	
I’ve been expressing my negative feelings.	
I’ve been trying to find comfort in my religion or spiritual beliefs.	
I’ve been trying to get advice or help from other people about what to do.	
I’ve been learning to live with it.	
I’ve been thinking hard about what steps to take.	
I’ve been blaming myself for things that happened	
I’ve been praying or meditating	
I’ve been making fun of the situation.	

Appendix D 

**Table 10:** Multidimensional Scale of Perceived Social Support (MSPSS) Reference: [16]

Items	Response
There is a special person who is around when I am in need.	
There is a special person with whom I can share joys and sorrows.	
My family really tries to help me.	
I get the emotional help & support I need from my family.	
I have a special person who is a real source of comfort to me.	
My friends really try to help me.	
I can count on my friends when things go wrong.	
I can talk about my problems with my family.	
I have friends with whom I can share my joys and sorrows	
There is a special person in my life who cares about my feelings	
My family is willing to help me make decisions.	
I can talk about my problems with my friends	
